# Relative safety of various spermatogenic stem cell purification methods for application in spermatogenic stem cell transplantation

**DOI:** 10.1186/s13287-019-1481-9

**Published:** 2019-12-16

**Authors:** Jia Tian, Ke Ma, Cheng-bin Pei, Shao-hua Zhang, Xue Li, Yue Zhou, Bei Yan, Hong-yan Wang, Liang-hong Ma

**Affiliations:** 1 General Hospital of Ningxia Medical University/Human Sperm Bank of Ningxia, Key Laboratory of Fertility Preservation and Maintenance of Ministry of Education, Ningxia Medical University, Yinchuan, 750001 China; 2 Clinical College, Ningxia Medical University, Yinchuan, 750001 China; 3 Key Laboratory of Fertility Preservation and Maintenance of Ministry of Education, Ningxia Medical University, Yinchuan, 750001 China

**Keywords:** Density gradient centrifugation, Fertility preservation, Flow cytometry, Immunomagnetic bead separation, Spermatogonial stem cell, Tumor

## Abstract

**Background:**

Spermatogonial stem cell (SSC) transplantation technology as a promising option for male fertility preservation has received increasing attention, along with efficient SSC purification technology as a necessary technical support; however, the safety of such application in patients with tumors remains controversial.

**Methods:**

In this study, we used a green fluorescent protein mouse xenograft model of B cell acute lymphocytic leukemia. We isolated and purified SSCs from the testicular tissue of model mice using density gradient centrifugation, immune cell magnetic bead separation, and flow cytometry. The purified SSCs were transplanted into convoluted seminiferous tubules of the nude mice and C57BL/6 male mice subjected to busulfan. The development and proliferation of SSCs in the recipient testis were periodically tested, along with whether B cell acute lymphocytic leukemia was induced following SSC implantation. The genetic characteristics of the offspring obtained from natural mating were also observed.

**Results:**

In testicular leukemia model mice, a large number of BALL cells infiltrated into the seminiferous tubule, spermatogenic cells, and sperm cells in the testis tissue decreased. After spermatogonial stem cell transplantation, the transplanted SSCs purified by immunomagnetic beads and flow cytometry methods colonized and proliferated extensively in the basement of the seminiferous tubules of mice; a large number of spermatogenic cells and sperm were found in recipient testicular tissue after 12 weeks of SSC transplantation. In leukemia detection in nude mice after transplantation in the three SSC purification groups, a large number of BALL cells could be detected in the blood of recipient mice 2–3 weeks after transplantation in the density gradient centrifugation group, but not in the blood of the flow cytometry sorting group and the immunomagnetic bead group after 16 weeks of observation.

**Conclusions:**

In this study, we confirmed that immunomagnetic beads and flow cytometry methods of purifying SSCs from the testicular tissue of the testicular leukemia mouse model could be safely applied to the SSC transplantation technology without concomitant tumor implantation. The results thus provide a theoretical basis for the application of tumor SSC cryopreservation for fertility preservation in patients with tumors.

## Introduction

Currently, about 1.7 in 1000 teenagers suffer from tumors including leukemia and testicular neoplasms, with a global incidence of pediatric tumors of 0.6% [1]. Notably, with the diversified development of tumor treatment technology (e.g., surgery, chemotherapy, radiotherapy, immunotherapy, and comprehensive treatment), the rate of treatment efficacy is increasing along with gradual annual increases in patient survival, allowing over 80% of patients to reach the criteria for recovery [2]. The therapeutic goal of tumor treatment has accordingly gradually shifted from a pursuit of recovery to survival quality, with fertility being an important factor that influences patient quality of life [3]. However, because the tumor itself as well as various factors during treatment affects fertility [4–6], 15% of cured patients will face infertility problems consequent to chemotherapy or radiotherapy that are both of a long duration [7] and accompanied by considerable psychological pressure [8]. Patients with tumors thus represent the main group applying for fertility preservation. Numerous fertility preservation technologies have therefore been developed including cryopreservation of spermatozoa, embryos, or testicular tissue, as well as spermatogonial stem cell (SSC) transplantation, which each exhibit respective advantages and disadvantages for the fertility preservation of male patients [9–12].

SSCs comprise male germline stem cells that are located in the basement membrane of the convoluted tubule of a testis and exhibit both self-renewal and directional differentiation potential. SSCs constitute the only self-renewing adult stem cells that can pass their genes to natural-born offspring throughout an animal’s lifespan [13]. SSC transplantation offers a unique clinical value for the fertility preservation of pre-pubescent patients with tumors [14]. The challenge of applying SSC transplantation in this scenario is to ensure successful separation and preservation of SSCs from testicular tissues that have potentially or actually been invaded by tumor cells, without causing tumor recurrence or reintroducing tumor tissues. However, although the most widely used extraction method can isolate and enrich SSCs from normal testicular tissues, numerous controversies remain regarding whether such obtained SSCs can be safely applied to the fertility preservation of patients with tumors.

To address the relative safety of various SSC isolation methods in a tumor background, in this study, we established a B cell acute lymphocytic leukemia (BALL) testicular leukemia model in mice and isolated and purified SSCs from the testis tissue of the model mouse using density gradient centrifugation, immunomagnetic bead-based cell sorting, or flow cytometry, and then carried out SSC transplantation. We then ascertained the fertility and offspring characteristics of the mice receiving the transplantation of the differently screened SSCs and whether any of the different SSC transplantation groups developed leukemia. This study will provide a theoretical basis for the safety and feasibility of SSC transplantation for the fertility preservation of patients with tumors.

## Materials and methods

### Ethics statement

Nude mice and C57BL/6 mice used in this study were purchased from the Animal Centre of Ningxia Medical University (Yinchuan, China). GFP mice were purchased from the Model Animal Research Center of Nanjing University (Nanjing, China). The experiment was approved by the Ethics Committee of Ningxia Medical University. Animal management during the experiment strictly abided by the *Guidance about Treating Experimental Animals* issued by the Ministry of Science and Technology of the People’s Republic of China in 2006. Human BALL-1 cells were purchased from ATCC.

### Construction of the nude mouse BALL model and observation of their survival state

Nude mice (male,4 weeks old) were randomly divided into eight groups (*n* = 10 mice per group): one normal control group and seven experimental groups that were injected with 10^2^–10^8^ human BALL cells via the caudal vein. The tumor model mice were executed by ip injection of pentobarbital (100 mg/kg) after the onset of cachexia. In addition, following BALL cell injection, one side of the testicular tissue was punctured every 7 days, with a total of three sites being punctured for smears and HE staining. BALL cells were detected until the testicular leukemia was diagnosed, and the onset time of testicular leukemia and the survival time of mice were recorded.

### Independent marker verification

The markers of BALL cells and mouse SSCs were determined through a literature review, with immunofluorescence and flow cytometry methods used to verify the independent expression of the respective markers in BALL cells and SSCs. Finally, CD20 and CD38 were chosen as BALL cell markers and CD90 and CD49f as an SSC marker for subsequent experiments.

### Immunofluorescence technique

Sections (5 μm) were baked in a constant temperature oven at 60 °C for 4 h, dewaxed by dimethyl benzene, and rehydrated by gradient alcohol. The tissue sections were washed with phosphate-buffered saline (PBS) (3 × 5 min, similarly hereinafter). The tissue was repaired using 0.01 M citrate buffer antigen for 15 min, cooled to room temperature, repaired once again, and rinsed thoroughly with PBS. The tissue sections were blocked with 5% bovine serum albumin (BSA) blocking solution at room temperature for 1 h and added with primary antibody liquid overnight at 4 °C (anti-CD20, Invitrogen, 14020982, 1:200; anti-CD38, Invitrogen, 14038982, 1:200; anti-CD90, Bioss, bs-0778R, 1:200; anti-CD49f, Abcam, ab105669, 1:200). The next day, the tissue sections were held at room temperature for 1 h then washed with PBS. The secondary antibody was added at room temperature for 1 h, followed by PBS washes. 4′,6-Diamidino-2-phenylindole (DAPI) was added for 30 min, then the tissue was rinsed with PBS. DAPI was added to seal the section, which was photographed using fluorescence microscopy.

### Immunohistochemistry technique

Sections (5 μm) were baked in a constant temperature oven at 60 °C for 4 h, dewaxed by dimethyl benzene, and rehydrated by gradient alcohol. The tissue sections were washed with PBS (3 × 5 min, similarly hereinafter). The tissue was repaired using 0.01 M citrate buffer antigen for 15 min, cooled to room temperature, repaired once again, and rinsed thoroughly with PBS. The tissue sections were blocked with 5% bovine serum albumin (BSA) blocking solution at room temperature for 1 h and added with primary antibody liquid overnight at 4 °C (anti-GFP, Proteintech, 66002-1-Ig, 1:200). The next day, the tissue sections were held at room temperature for 1 h then washed with PBS. The secondary antibody was added at room temperature for 1 h, followed by PBS washes. Hematoxylin was added for 3 min, then the tissue was dehydrated using graded ethanol, vitrification by dimethylbenzene, and seal sheet with neutral gum and photographed using microscopy.

### Flow cytometry detection

Mouse blood (50 μL) in 2% EDTA as a coagulant was added to 1 mL PBS and centrifuged three times at 4 °C, 300*g* for 5 min. PBS (500 μL) was added to the pellet for re-centrifugation. Primary antibody was added (anti-CD20, Invitrogen, 14020982, 1:100; anti-CD38, Invitrogen, 14038982, 1:100; anti-CD90, Bioss, bs-0778R, 1:100; anti-CD49f, Abcam, ab105669, 1:100, anti-UTF1, Thermo Fisher, MFCDA84, 1:250; anti-PLZF, Bioss, Bs5971R, 1:250; CD117, Bioss, Bs20716R, 1:200), then cells were incubated at 4 °C for 1 h, washed with PBS, and centrifuged as described above. Fluorescent secondary antibody was added, and cells were incubated at 4 °C for 1 h, then centrifuged as above, except with the addition of 1 mL PBS for re-centrifugation. Cells were filtered through a 40-μm mesh and tested by an instrument (DM2000, Leica, USA).

### Extraction and purification of SSCs

Following confirmation of the successful construction of the testicular leukemia model, mouse testicular tissue was collected under aseptic condition and the albuginea was stripped. The tissue was cut into 2–3-mm pieces, to which five volumes of 0.5% trypsin were added for digestion. When the tissues were digested to single cells, 2 mL serum was added to stop the digestion. The cells were centrifuged at 300*g* for 5 min, collected, washed with PBS three times, and filtered through a 40-μm mesh. The filtrate was collected, and SSCs were isolated and purified using the following methods according to each group.

### Density gradient centrifugation for SSC screening

Percoll density gradient liquid was prepared in the order of 20%, 30%, 35%, 40%, 45%, 50%, and 60%. A 1-mL aliquot of each gradient was absorbed and added to a 15-mL centrifuge tube according to the gradient from large to small. A straw was used to carefully move the cell suspension to be separated, which was added on the separation medium at the top of the gradient fluid in the 15-mL centrifuge tube. The centrifuge tube was centrifuged at 4 °C, 500*g* for 10 min, and cells from the 35–45% gradient were collected, washed with PBS with three centrifugation steps at 300*g* for 5 min. The supernatant was abandoned, and the cells were re-suspended with 1.5 mL PBS. Cell count was determined, and the cell concentration was adjusted to 10^7^/mL.

### Immunomagnetic bead-based sorting for SSC screening

Cells of the obtained testicular cell suspension were re-suspended with magnetic bead separation buffer and precipitated into a centrifuge tube to obtain cell count. After centrifugation at 300*g*, the supernatant was abandoned. Antibody solution was added to the cells following instructions (CD20 microbeads, MACS, 130093452; CD38 microbeads, MACS, 130092263; CD90 microbeads, MACS, 12000295; anti-CD49f, Abcam, ab105669; anti-rat IgG microbeads, Miltenyi Biotec,130048502), incubated at 4 °C for 30 min after mixing, centrifuged at 300*g*, and washed three times. Then, 500 μL separation buffer was added to the final pellet to re-suspend the cells. A new centrifuge tube was placed into the grooves of the magnetic frame, and a pipette was used to carefully add the cell suspension. Magnetic bead separation buffer (500 μL) was added before the last drop of the filtrate, for a total of three times. Then, the cell separation tube was immediately removed from the magnetic field, and 1 mL of separation buffer was added, for a total of three times. Cells were centrifuged at 300*g* and counted, and the cell concentration was adjusted to 10^7^/mL.

### Flow cytometry for SSC screening

Cells after suspension and filtration were centrifuged, and the supernatant was abandoned. Primary antibody 200 μL (anti-CD20, Invitrogen, 14020982, 1:100; anti-CD38, Invitrogen, 14038982, 1:100; anti-CD90, Bioss, bs-0778R, 1:100; anti-CD49f, Abcam, ab105669, 1:100) was added to the pellet, mixed with cells, incubated at 4 °C for 30 min, centrifuged at 300*g*, and washed three times with PBS. Then, 200 μL FITC-labeled fluorescent antibody (1:100) was added, and cells were incubated at 4 °C for 30 min, centrifuged at 300*g*, washed three times in PBS, and screened by flow cytometry (FACS Vantage SE, BD Biosciences, Canada). Cells were screened and counted, and the cell concentration was adjusted to 10^7^/mL.

### Preparation of SSC transplant recipient mice and SSC transplantation

SSC transplant recipient mice (male, 4 weeks old) and SSC transplantation refer to the articles published by the research group [15], the incision was disinfected with alcohol and covered with iodine volts gauze and an elastic bandage. The skin incision was disinfected once daily. Penicillin was administered at 50,000 U/mouse via ip injection, for three successive days.

### Western blotting

Tissues and cells were collected under aseptic condition and lysed with RIPA buffer. The protein concentration was determined using the BSA method after extraction according to the kit instruction (KGPBCA, Keygen Biotech, Nanjing China). Proteins were separated using 4–15% sodium dodecyl sulfate-polyacrylamide gel electrophoresis, transferred onto polyvinylidene fluoride membranes, blocked with 5% skim milk powder for 1 h, then the primary antibody was added (anti-CD20, Invitrogen, 14020982, 1:2000). The next day, the membrane was kept at room temperature for 1 h and washed with PBS for 3 × 5 min. Corresponding secondary antibody (Proteintech, Rosemont, IL, USA) was added. The membrane was incubated at 37 °C for 1 h and washed with PBS. ECL was added and imaged by an instrument (ChemiDoc XRS^+^, Bio-Rad, Canada).

## Results

### Detection of independent markers in BALL cells and testis SSCs

**Fig. 1:**
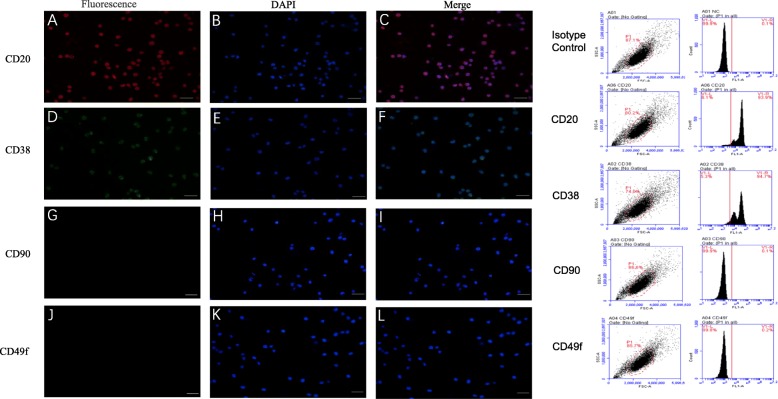
Marker verification and validation of independent markers in BALL cells by immunofluorescence and flow cytometry. The expression of CD20 (**a**–**c**) and CD38 (**d**–**f**), were positive on BALL cell membranes. CD90f (**g**–**i**) and CD49f (**j**, **k**) were not expressed in BALL cells. DAPI indicates the cell nucleus. This finding was confirmed via the conducted flow cytometry analysis. Scale bar = 100 μm

**Fig. 2:**
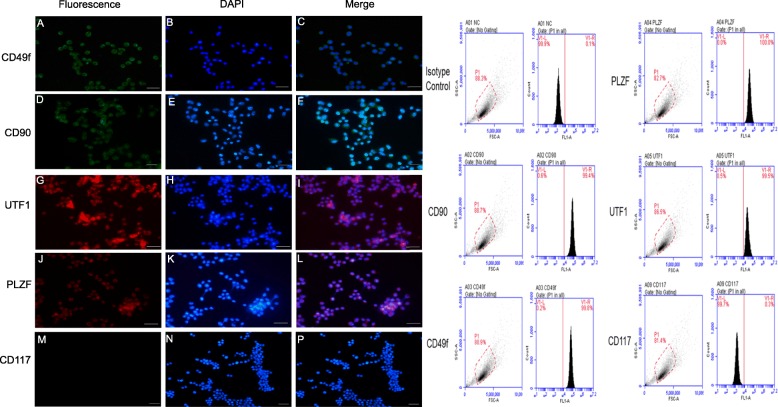
Marker verification and validation of independent markers in SSCs by immunofluorescence and flow cytometry. Undifferentiated SSCs expressed CD49f (**a**–**c**), CD90 (**d**–**f**), UTF1 (**e**–**f**), and PLZF (**j**–**l**), but not CD117 (**m**–**p**). DAPI indicates the cell nucleus. This finding was confirmed via the conducted flow cytometry analysis. Scale bar = 100 μm

**Fig. 3:**
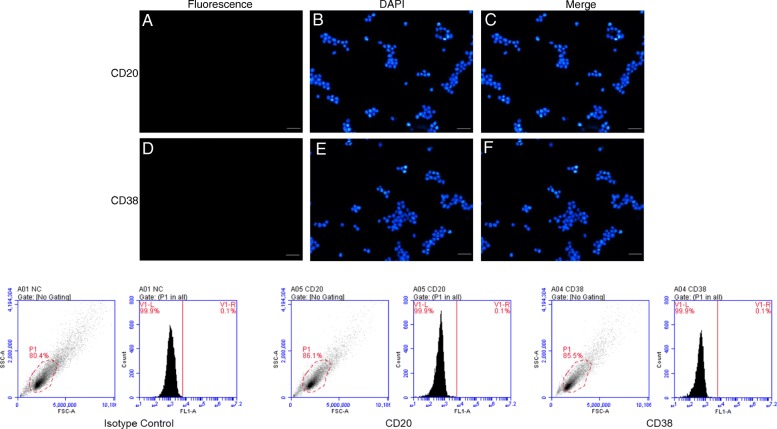
CD20 and CD38 verification and validation of independent markers in SSCs by immunofluorescence and flow cytometry. CD20 (**a**–**c**) and CD38 (**d**–**f**) were not expressed in SSCs. DAPI indicates the cell nucleus. This finding was confirmed via the conducted flow cytometry analysis. Scale bar = 100 μm

Currently, among the known BALL cell expression markers, CD20 and CD38 were positive on BALL cell membranes, the positive rate of CD20 and CD38 is greater than 90%, and CD49f and CD90 were not expressed in BALL cells (Fig. 1). over 20 types of SSC, markers have been identified. CD49f, CD90, UTF1, and PLZF were expressed in undifferentiated SSCs whereas CD117 was expressed in differentiated SSCs (Fig. 2) and CD20 and CD38 were not expressed in SSCs (Fig. 3). Therefore, we used CD49f and CD90 as the markers for SSC sorting in this study in order to improve the purity of purified SSCs.

### Establishment and characterization of the testicular leukemia model including BALL cell detection in nude mice

**Fig. 4:**
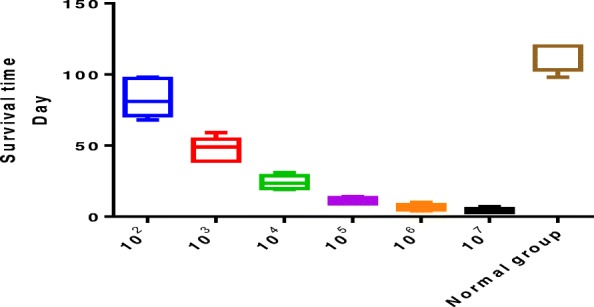
Onset and survival of nude mice. Onset and survival time of leukemia after different numbers of BALL cells were injected into the tail vein of nude mice

The overall design of the study is shown in the flowchart. To establish a testicular leukemia model, different doses of human BALL cells were injected into nude mice by tail vein injection; The onset time of leukemia in each group of nude mice was recorded (Fig. 4). With the increase in the number of injected cells, the onset time and survival time of mice gradually decreased.

**Fig. 5:**
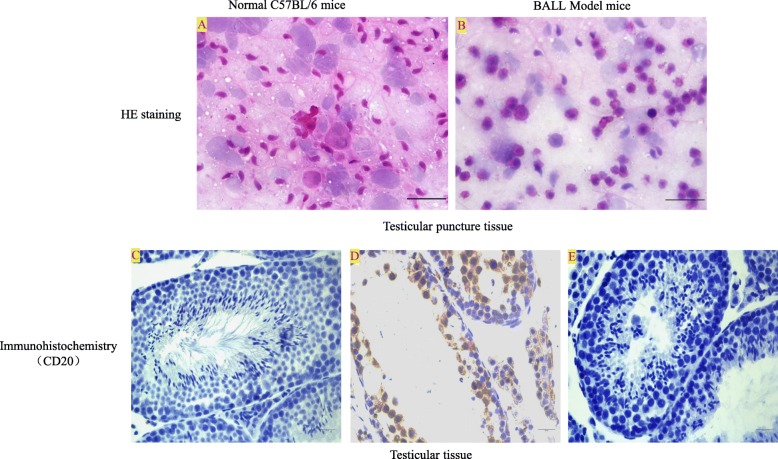
Detection of testicular leukemia model mice. HE staining: **a** In normal C57BL/6 mice, a large number of spermatogenic cells and sperm cells could be seen in testis tissue. **b** In the model group, after 10^4^ cells were injected to establish the model for 3 weeks, spermatogenic cells and sperm cells in the testis tissue decreased with the infiltration of BALL cells. Immunohistochemistry: **c** In normal C57BL/6 mice, the expression of CD20 protein in testicular tissue was negative. **d** In the model group, a large number of CD20-positive BALL cells infiltrated into the seminiferous tubule, spermatogenic cells and sperm cells in the testis tissue decreased. **e** Negative control. Scale bar = 100 μm

All models were successful, and the nude mice exhibited severe BALL and cachexia symptoms; the testicular tissue was collected aseptically. HE staining and immunohistochemistry were used to detect BALL cells (Fig. 5). In the model group, after 10^4^ cells were injected to establish the model for 3 weeks, a large number of CD20-positive BALL cells infiltrated into the seminiferous tubule, spermatogenic cells, and sperm cells in the testis tissue decreased. A testicular leukemia model was also generated using GFP mice, using similar methods; subsequently, the GFP^+^ SSCs were separated and purified.

### SSC transplantation and GFP detection

**Fig. 6:**
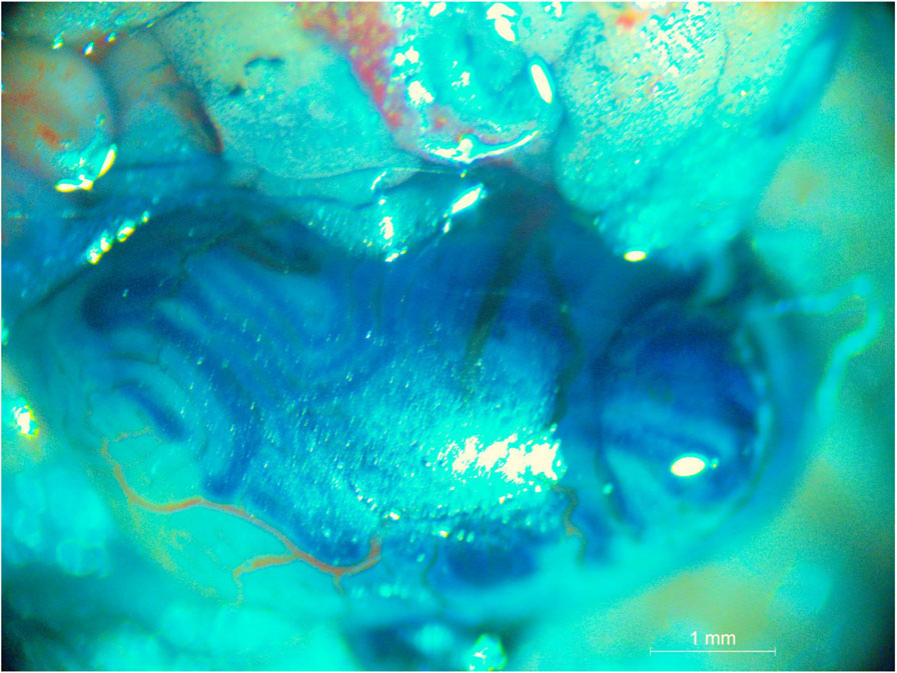
Transplantation of SSCs. Trypan blue staining was used to show the SSC transplantation procedure. A 3-mm incision was made at the dorsal tip of the testis tissue of the recipient mouse, and the seminiferous tubules were exposed. After the injection, the trypan blue solution gradually filled along the seminiferous tubules. One injection (40–50 μL) could fill one fourth to one third of the testicular tissue. Scale bar = 1 mm

**Fig. 7:**
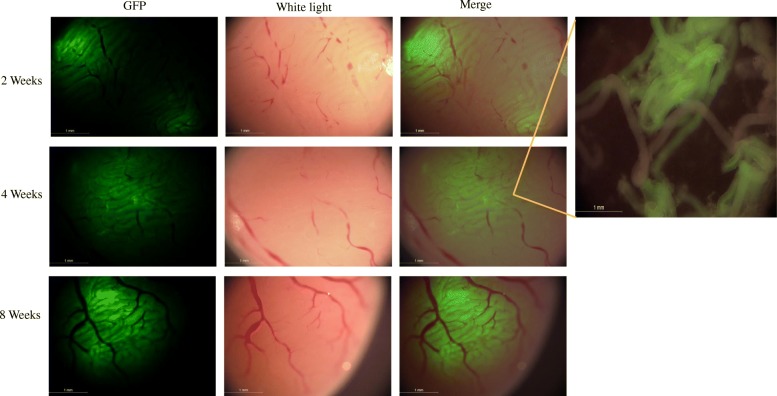
Proliferation and differentiation of GFP mouse SSCs after transplantation. The GFP-positive volume in the testis of the recipient mice gradually expanded over time, and extensive GFP expression could be observed in the testis. Scale bar = 1 mm

**Fig. 8:**
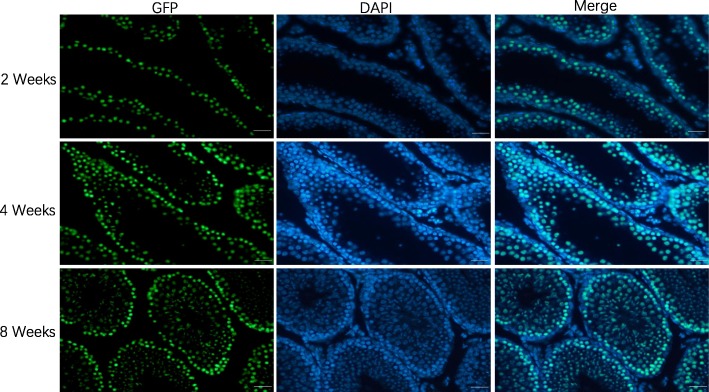
Proliferation and differentiation of SSCs after transplantation. Paraffin sections were prepared from the transplanted mouse testes. Immunofluorescence was used to detect the colonization, proliferation, and differentiation of the transplanted SSCs in the seminiferous tubules of the mice. The transplanted SSCs colonized and proliferated extensively in the basement of the seminiferous tubules of mice and differentiated into spermatogenic cells at various levels. Scale bar = 100 μm

SSCs that were isolated and purified by each of the three methods were respectively transplanted into the seminiferous tubules of 4-week-old nude mice and C57BL/6 mice. Figure 6 shows the SSC transplantation procedure. After transplantation, extensive GFP expression in recipient mouse testis suggested that the GFP-positive SSCs extensively proliferated and differentiated in the seminiferous tubules of the recipient mice, and differentiated into spermatogenic cells at various levels (Figs. 7 and 8).

### Epigenetic characteristics of SSC-transplanted mouse progeny

**Fig. 9:**
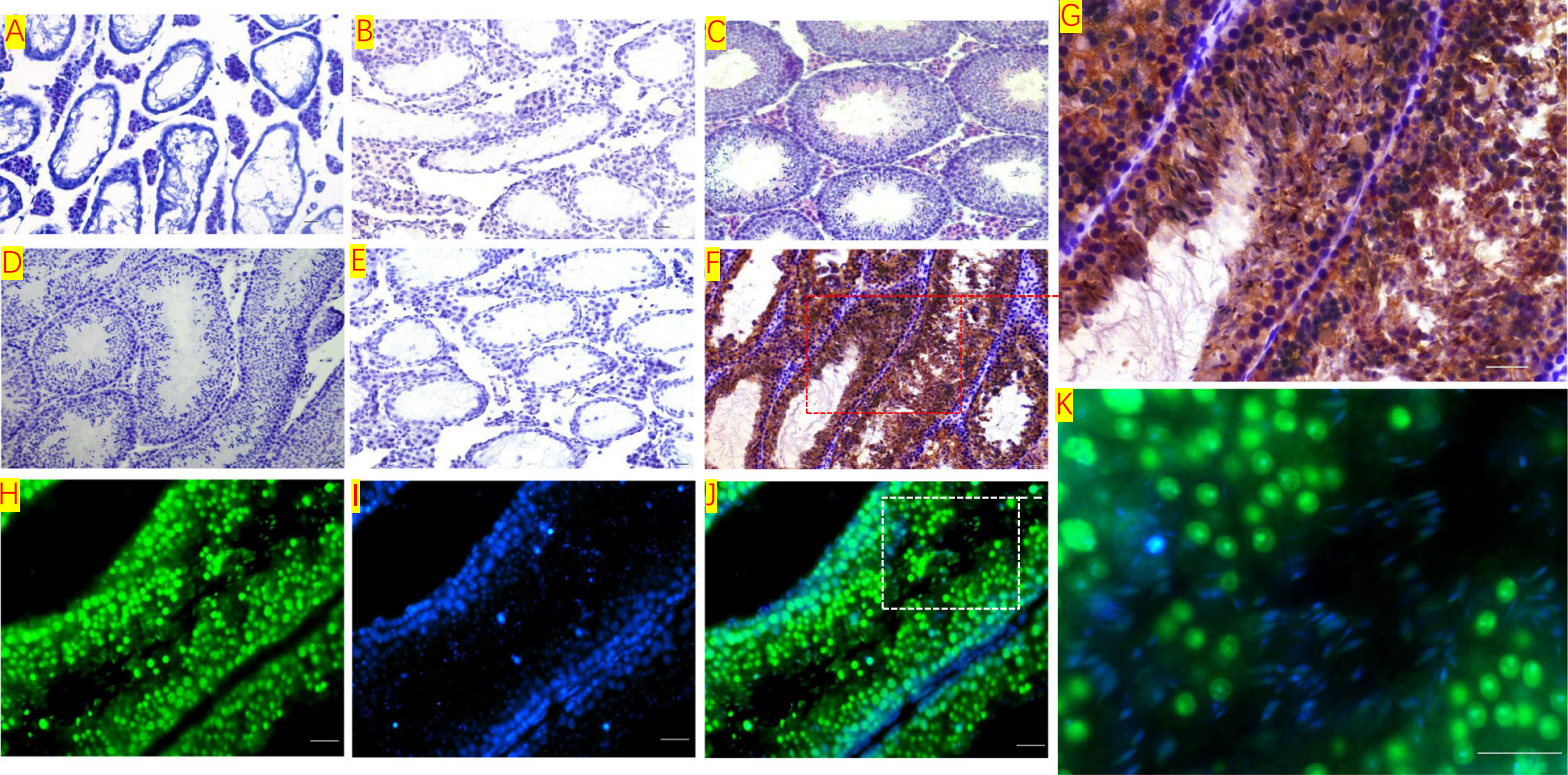
Morphological changes of testicular tissue before and after transplantation. **a** Testicle of SSC transplantation recipient; it can be seen that spermatogenic cells in testicular tissue disappeared. **b** After 12 weeks of natural recovery, the spermatogenic function was still not recovered. **c** A large number of spermatogenic cells and sperm were found in recipient testicular tissue after 12 weeks of SSC transplantation. **d**–**g** Immunohistochemical staining for detection of GFP expression in testicular tissues. **d** Negative control. **e** Natural recovery of testicular tissue in mice after 12 weeks of treatment with busulfan. **f**, **g** Testicular tissue after 12 weeks of GFP+ SSC transplantation, a large number of GFP+ spermatogenic cells and spermatozoa in the convoluted tubule. Immunofluorescence assay staining for detection of GFP expression in testicular tissues after 12 weeks of SSC transplantation (**h**–**k**). **h** GFP. **i** DAPI. **j**, **k** Meger. Magnification, scale bar = 100 μm

**Fig. 10:**
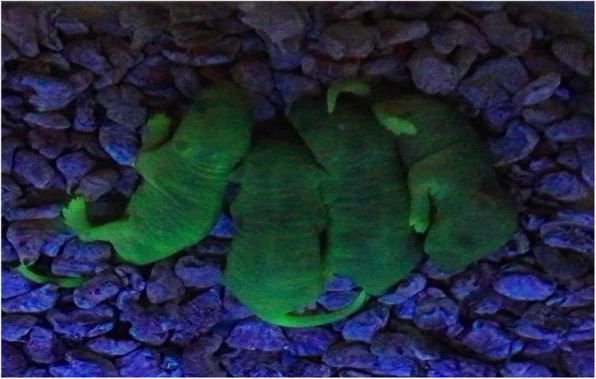
Fecundity of semen GFP sperm. Detection of epigenetic characteristics of the progeny of SSC transplantation mice: C57BL/6 mice after SSC transplantation and 4-week-old female C57BL/6 were bred at 1:2 to observe the epigenetic characteristics of the progeny as assessed by GFP expression. The offspring that were born by natural mating of the GFP-transplanted mice and the C57 mice expressed GFP protein, which emitted green fluorescence under UV excitation

Epigenetic characteristics of recipient mouse spermatozoa after SSC transplantation, HE staining, immunohistochemistry, and immunofluorescence detected in the testicle of SSC transplantation can be seen with a large number of GFP+ spermatogenic cells, and sperm were found in recipient testicular tissue after 12 weeks of SSC transplantation; on the contrary, only a few spermatogenic cells but no mature spermatozoa were observed in the tubules of mice after natural recovery for 12 weeks (Fig. 9). Normal fertilization by transplanted GFP+ SSC-derived sperm was confirmed by the generation of progeny following mating with C57BL/6 female mice (Fig. 10).

### Detection of leukemia after GFP SSC transplantation into nude mice

**Fig. 11:**
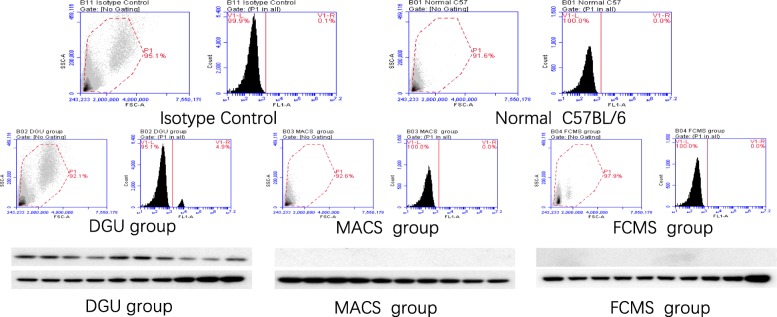
Leukemia detection in nude mice after transplantation in the three SSC purification groups as assessed by blood flow cytometry and western blotting. A large number of BALL cells could be detected in the blood of recipient mice 2–3 weeks after transplantation in the density gradient centrifugation (DGU) group, but not in the blood of the flow cytometry sorting (FCMS) group and the immunomagnetic bead (MACS) group after 16 weeks of observation. CD20 detection in testicular tissue of nude mice after transplantation in the three groups by western blotting. High CD20 expression was detected in testicular tissue of nude mice in the density gradient centrifugation (DGU) group, but not in the flow cytometry sorting (FCMS) and immunomagnetic bead sorting (MACS) groups after 16 weeks of observation

At the end of the observation, high expression of CD20 was detected in the blood and testicular tissue of the recipient nude mice in the density gradient centrifugation group, but not in the blood from nude mice in the flow cytometry and immunomagnetic bead-based selection groups (Fig. 11). Overall survival for each group in the two mouse strains was given in Tables 1 and 2.

**Table 1:** Statistics of survival status of nude mice after transplantation with SSCs purified by each of the three methods (mean ± SD)

Group (nude mouse group)	Density gradient centrifugation group (*n* = 10)	Flow cytometry sorting group (*n* = 10)	Immunomagnetic bead sorting group (*n* = 10)
Number of cases of leukemia	10 (100%)	0	0
Average number of days of leukemia	12.5 ± 4.3	–	–
Average survival time	22.5 ± 4.6	111.0 ± 12.5	117.6 ± 6.9
Number of cases detected of testicular GFP sperm	0	10 (100%)	10 (100%)
Average days of testicular GFP sperm	–	49.7 ± 5.8	47.6 ± 6.7

**Table 2:** Survival status and progeny statistics of C57BL/6 mice after transplantation with SSCs from each of the three purification methods (mean ± SD)

Group (C57BL/6 group)	Density gradient centrifugation group (*n* = 10)	Flow cytometry sorting group (*n* = 10)	Immunomagnetic bead sorting group (*n* = 10)
Number of cases of leukemia	10 (100%)	0	0
Average number of days of leukemia	18.2 ± 3.6	–	–
Average survival time	30.9 ± 4.8	113.1 ± 12.2	114.4 ± 11.3
Number of cases of testicular GFP sperm detected	0	10 (100%)	10 (100%)
Average detection time of testicular GFP sperm	–	50.1 ± 4.6	42.8 ± 6.0
Number of births of offspring GFP mice	0	8 (80%)	10 (100%)
Number of births of offspring non-GFP mice	0	0 (0%)	0 (0%)

## Discussion

The overall incidence of tumors as well as that of tumors occurring in younger people is increasing worldwide, although treatment advances have concomitantly improved survival rates. The incidence of childhood tumors is 1.1%, and the incidence of adolescent tumors is 2% [16]. A study published in the USA targeting 380,000 children with tumors indicated 5-, 20-, and 60-year survival rates of 83.5%, 44.9%, and 5%, respectively, with the 60-year survival rate reaching approximately 15% for cases such as germ cell, trophoblastic, and gonadal tumors (17.7%) and soft tissue tumors (14.6%) [17]. As fertility is critical for maintaining a normal quality of life in adulthood, the preservation of fertility for this population represents the predominant application for related technologies.

Male fertility preservation is currently effected via gamete or testicular/ovarian tissue cryopreservation, or SSC preservation and transplantation. Alternatively, embryo cryopreservation requires the provision of mature germ cells and in vitro fertilization to form an embryo, followed by cryopreservation. Although cryogenic sperm technology itself is relatively mature and the clinical effect of post-resuscitation fertility is relatively certain, the application is restricted for immature children and those unable to provide sperm. In turn, whereas testicular tissue can be frozen and then revived, its transplantation technology is not yet mature and the effects of cryogenic resuscitation markedly differ based on specimen size, patient age, and other factors [18]. Moreover, there is also the risk of tumor re-implantation in patients with cancer. In contrast, SSC cryotransplantation technology has a wide range of application prospects in fertility preservation, especially for sexually immature children and patients with limited use of other technologies [19], thereby providing the best research potential for fertility preservation. In particular, a mean biopsy size of only 0.34 mm^3^ would maximally result in 14 × 10^5^ type A dark spermatogonia collected [20], which satisfies the requirement for fertility preservation. Thus, the safety of SSC transplantation technology for the fertility preservation of patients with tumors is worthy of in-depth investigation, with the primary question of whether it can successfully separate and purify SSCs without tumor cells from testicular tissue.

Intravenous injection of leukemia cells is currently widely used to establish leukemic model mice, affording the advantages of simple operation and high success rate [21]. To develop a mouse model of BALL, in the present study, we first validated human BALL cell-specific detection markers, CD20 and CD38, by immunofluorescence and western blotting and found that CD20 and CD38 were widely expressed in BALL cells whereas the SSC markers CD90 [22–24] and CD49f [14] were not expressed. We then injected the human BALL cells into 4-week-old nude mice via tail vein injection to establish the BALL model mice. Notably, as the number of cells injected increased, leukemia onset and survival time of mice were accordingly shortened.

Obtaining highly purified SSCs from donor testis tissue constitutes the primary issue for SSC transplantation technology. The currently widely used methods for extracting spermatogonial stem cells are differential adherence, Percoll density gradient centrifugation [25], and flow cytometry [15] or immunomagnetic bead-based sorting [26], which can all satisfactorily separate and purify SSCs from testicular tissue capable of successfully restoring male fertility [20, 27, 28]. In previous studies, we had demonstrated that SSC transplantation restored spermatogenesis to busulfan-treated mice with azoospermia [15, 29, 30]. In the present study, we modeled azoospermic SSC transplant recipient mice through busulfan treatment; we found that the transplanted GFP+ SSCs extensively proliferated and differentiated in the testis tissue of the recipient mice and eventually formed mature spermatozoa over time. GFP+ spermatozoa were first detected in the recipient mouse testis as early as 32 days after transplantation with an average time of 47.6 ± 16.4 days post-transplantation.

We further observed the incidence of leukemia in nude mice by transplanting SSCs that had been isolated and purified through either of the three methods (density gradient centrifugation, flow cytometry, or immunomagnetic bead-based sorting) into the seminiferous tubules. It was found that all the nude mice receiving density gradient centrifugation-purified SSCs developed leukemia symptoms after transplantation, with the earliest detection time of 11 days after transplantation. The detection of peripheral blood BALL cells by flow cytometry and as well as CD20 and CD38 expression in testicular tissue together indicated that BALL cells had invaded the testicular tissue. Moreover, no GFP+ spermatozoa were detected in the testis of these nude mice, suggesting that the spermatogenic function of testicular tissue invaded by BALL cells was impacted. However, none of the nude mice receiving SSCs purified by immunomagnetic beads and flow cytometry developed symptoms of leukemia, and no BALL cells were detected in the blood and testis tissues of recipient nude mice by flow cytometry. This indicated that these two methods of purifying SSCs from the testicular tissue of the testicular leukemia mouse model could be safely applied to the SSC transplantation technology without concomitant tumor implantation. Previous studies showed that the receipt of 20 leukemia cells in mouse testicular tissues would result in the tumor onset of recipient mice [31]. In particular, Hou et al. [32] used surface markers to identify leukemic (CD4 and major histocompatibility complex class I) and germ (epithelia cell adhesion molecule) cells in testicular samples infiltrated with Roser’s T cell leukemia. These markers were then used to delete leukemic cells and/or select for germ cells by flow cytometry, which demonstrated that flow cytometric purification of germ cells from a leukemic donor is not highly effective or safe for clinical use. Hu et al. [33] pointed out that the major limiting factor for flow cytometry-based purification is the low detection level of contaminating cancer cells, which at its best is one event among 10^3^–10^4^ sorted cells. In contrast, Hermann et al. [34] sorted SSCs from the testis of patients with T lymphoblastic leukemia by flow cytometry using the CD90+/CD45− standard, which showed that SSCs sorted by flow cytometry were safe for transplantation, although purity testing was required prior to transplantation. Fujita et al. [31] also succeeded in separating leukemia tumor cells by flow cytometry using MHC class I and CD45 as markers. Notably, however, the expression of various markers differed owing to the difference of leukemia tumor cell types [35, 36], and the expression rate of any marker on tumor cells was not 100% [35–41]. Therefore, our results suggest that the use of a single marker in such screening is not sufficient, whereas success may be more readily achieved through the use of a combination of multiple screening markers.

## Conclusions

In this study, we confirmed that immunomagnetic beads and flow cytometry methods of purifying SSCs from the testicular tissue of the testicular leukemia mouse model could be safely applied to the SSC transplantation technology without concomitant tumor implantation. The results thus provide a theoretical basis for the application of tumor SSC cryopreservation based on the two sorting-based SSC purification methods for fertility preservation in patients with tumors.

## Data Availability

The original data are available from the corresponding author on request.

## Abbreviations

ATCC: American Type Culture Collection
BALL: B cell acute lymphocytic leukemia
DAPI: 4′,6-Diamidino-2-phenylindole
DGU: Density gradient centrifugation
FCMS: Flow cytometry sorting
GFP: Green fluorescent protein
H&E: Hematoxylin and eosin
MACS: Magnetic activated cell sorting
SSC: Spermatogonial stem cell
UV: Ultraviolet lamp
